# The Relation of the Nasal Mucosa to the Genitals

**Published:** 1897-09

**Authors:** Byron Robinson

**Affiliations:** Chicago, Professor in the Chicago School of Gynecology and Abdominal Surgery; Professor of Gynecology in the Harvey Medical College and the Illinois Medical College; Gynecologist to the Woman’s Hospital; Gynecologist to the Woman’s Charity Hospital and Consultant Surgeon to the Mary Thompson Hospital for Women and Children


For the Texas Medical Journal.

### The Relation of the Nasal Mucosa to the Genitals.

BY BYRON ROBINSON, B. S., M. D., CHICAGO.

Professor in the Chicago School of Gynecology and Abdominal Surgery; Professor of Gynecology in the Harvey Medical College and the Illinois Medical College; Gynecologist to the Woman's Hospital; Gynecologist to the Woman's Charity Hospital and Consultant Surgeon to the Mary Thompson Hospital for Women and Children.

IT IS A CURIOUS FACT that even laymen have for ages noted that the organ of smell is closely related to the generative organs, but it is very recently that specialists (gynecologists and rhinologists are putting together the connected story. The relation and connection of the olfactory organ and nasal mucous membrane with the genitals are by way of the sympathetic. The anatomic paths of travel from the nasal mucous membrane to the genitals are by way of the fifth cranial nerve or trigeminus—a sympathetico-cranial nerve. The trigeminus is supremely the ganglionic cranial nerve. It is the type of mixed nerves. It has eight ganglia situated on its branches. It also sends a large branch to the mucous membrane of the nose—the nasal nerve. This will at once explain its wide influence in reflection of disease, because of its extensive influence over the calibre of the adjacent blood and lymph vessels and the extensive periphery in the nasal mucosa, allowing opportunity for considerable reflexes.

Let us examine for a moment the ganglia of the trigeminus (tri-facial) or fifth cranial nerve—the ganglionic nerve of the brain. A significant statement may precede the short description by saying that one of the chief offices of a ganglion is to demedullate nerves.

1. We may note the Gasserian ganglion of the fifth cranial nerve situated in a depression in the apex of the petrous portion of the temporal bone. It is as large as the end of the little finger. The ganglionic nature of this swelling was first perceived by Raimund Balthessar Hirch, a Vienna anatomist, in 1765, who christened it the "ganglion Gasseri," in honor of his teacher, Johann Laurentius Gasserius, who in 1779 was "Privet Docent" in anatomy under Prof. Joseph Jaus, in Vienna. Since DuBois-Raymond announced from personal experience, that he thought facial neuralgia was "due to spasmodic contraction of the blood vessels controlled by the sympathetic," surgeons have attempted to cure facial neuralgia by destruction of J. L. Gasser's ganglion. This long continued attempt by surgeons at the cure of facial neuralgia, is a recognition at least, of the sympathetic nature of the Gasserian ganlion and its consequent influence over the calibre of blood vessels of the same side of the face.

The Gasserian ganglion has close and intimate connection with the sympathetic nerves. The blood vessels alone which are necessary to supply the Gasserian ganglion, would produce a close and intimate relation with the sympathetic and tri-facial. The trigeminus nerve shows a very intimate and extensive connection with the sebaceous glands of the face and the genitals. This is seen at puberty, of both boys and girls (facial acne), and in the menopause. The changes in voice of boys at puberty, and the changes of voice of women at the monthly, may be easily worked out anatomically by dissecting out the connection between the superior cervical ganglion and the pneumogastric and glosso-pharyngeal. Also the spheno-palatine ganglion sends branches to the tonsils in the descending palatine nerves. One may find three to five branches of nerves passing from the superior cervical ganglion to the glosso-pharyngeal and pneumogastric nerves. At the menstruation the vocal chords are congested, and hence the hoarse, husky voice and similar permanent physiologic process of congestion and growth occurs in the boy at puberty. Hence, the close and intimate relations of the vocal chords (voice) and nasal mucosa (smell and reflex action)

with the genitals have a distinct, concrete, anatomical explanation. Besides the larynx is supplied by the sympathetic branches which accompany the superior and inferior recurrent laryngeal nerves.

2. The ophthalmic, lenticular or ciliary ganglion is a pinhead sized ganglion situated in the orbit. It is closely connected by roots to the nasal branch of the fifth nerve, i. e., has relations with the nasal mucosa, by a sympathetic branch from the cavenous plexus. It is also connected with the third cranial. This second ganglion has intimate connection with the nasal mucosa.

Joseph Guischard Duverney (1648-1730), a French anatomist, discovered this ganglion.

3. The spheno-palatine, or Meckel's ganglion, situated in the spheno-palatine fossa and on the superior maxillary branch of the tri-facial is a large mass of nerve cells. It is intimately connected with the nasal mucosa by the descending palatine nerves. The spheno-palatine ganglia was discovered and described by Johan Freiderich Meckel (1717-1774), a celebrated German anatomist. Like all the other ganglia of the fifth cranial nerve, it possesses a motor, sensory and sympathetic root. It sends considerable nerve supply to the tonsils. Hence, we again observe that that ganglion shares in distributing nerves to the nasal mucosa and the region of the tonsils. But the premise of our argument is that the fifth nerve being studded by eight sympathetic ganglia is intimately and closely connected anatomically and functionally with the genitals. Therefore, what affects the fifth nerve will affect the genitals and *vice versa*.

4. The ottic or Arnold's ganglion is located just below the foramen ovale, on the inferior maxillary branch of the trifacial. Its sympathetic branches are derived from the sympathetic plexuses which surround the adjacent middle meningeal artery. It is connected with the facial and glosso-pharyngeal nerves and sonds branches to the tensor palati. In our library may be seen Friedrich Arnold's "Anatomie des Menschen," 3 volumes. On page 909, volume 2, Arnold says: "Der Ohrknoten wurde von *im* Winter 1825-26 endeckt." (In English, The ottic ganglion was discovered by me in the winter of 1825-26.) Professor Arnold noted seventy-five years ago that many tried in vain to show that others than himself discovered the ganglion. This ganglion shows connection with the larynx by way of the glossopharyngeal and tensor palati and through the vidian nerve and Meckel's ganglion with the nasal mucosa.

5. The submaxillary ganglion is situated on the lingual branch of the inferior branch of the trifacial nerve. Its sympathetic branch is derived from the plexus which surrounds the adjacent facial artery. This ganglion was discovered by Johann Friederich Meckel (1717–1774) in 1748. It has been named after him, "ganglion Meckelli minus." The ganglion communicates with the facial or the seventh nerve.

6. The sublingual or Blandin's ganglion is situated on the branch of nerves to the sublingual gland. This collection of nerves may be only a plexus or a ganglion. It will have similar connection to the submaxillary ganglion. Phillippe Frederic Blandin (1798–1849), a French surgeon, first described this ganglion in 1840.

7. The ganglion of Bochdelek is located at the junction of the middle superior dental nerve with the anterior superior dental nerve. It is not constant, and, besides, the swelling may not always be a ganglion, i. e., may not contain nerve cells. It lies above the upper canine tooth. Victor Alexander Bochdalek (father), Professor of Anatomy in Prague until 1869 (papers published in 1866). Victor Bochdalek (son), also an anatomist in Prague. However, it appears to be the father who discovered this ganglion, for I find in Arnold's anatomy that Bochdalek had observed this ganglion previous to 1851.

8. The ganglion of Valentine is situated at the junction of the middle superior dental with the posterior superior dental nerve. It is located above the second bicuspid tooth. The ganglion was discovered by Gabriel Gustave Valentine (1810–1883), a German anatomist. All the ganglia of the fifth cranial or trifacial have a sympathetic connection.

We have shown, first, that the trigeminus is a supremely ganglionic cranial nerve; that it is closely and intimately connected with the genitals by way of sympathetic tracts; also that the trigeminus is closely and intimately connected, especially with the nasal mucosa, and to a considerable extent, with the larynx nnd vocal chords. There is found to be numerous and intimate connection between the fifth cranial nerve, the trigeminus and the seventh cranial nerve, the facial. Observation shows the intimate and close relations of the genitals with the voice, nasal mucosa and the facial sebaceous gland at puberty and menstruation. This close connection and intimate relation is accomplished by means of the sympathetic nerves, especially the ganglia on the trifacial. This physiologic relation of the genitals to the trifacial and facial nerves may be plainly observed in the sexual relations and cohabitations of animals.

Irritation of the nasal mucosa will cause congestion and erection. Occasionally irritation of the genitals will cause congestion of the face or region of the trigeminus. Urethral irritation will induce "gritting" of the teeth, i. e., action of the masseter muscles supplied by the inferior branch of the fifth.

Dr. A. G. Hobbs describes two cases of severe priapism accompanying acute rhinitis. (Jr. Amer. Med. Asso., 1897.) On spraying the nasal mucosa with cocaine the priapism immediately subsided. Opium affected the priapism in each case, but only to a slight degree.

A reflex sneeze is not infrequent previous to erection. In preparations for coition the involvement of the nasal mucosa is quite apparent in animals, as the horse, dog, bull, etc. In monkeys the nasal mucosa is not only involved in coition, but it is evident the larynx is highly involved, from the active and vigorous chattering, emitted previous to and during coition. The mare neighs with the approaching stallion, the growling of dogs, noise of cats and cackling of hens is doubtless not accidental at times of coition, but is due to irritation of nerve tracts.

The tissue covering the turbinated bones is quite erectile. A nasal reflex will induce an erectile action in the corpora cavernosa. We know that the genitals are intimately and profoundly supplied by the sympathetic nerves; we know that the fith nerve is the supremely ganglionic (sympathetic) nerve of the brain. The fifth nerve sends a rich nerve supply to the nasal mucosa and to the larynx through the vagus and glosso-pharyngeal. The facial and trigeminus are intimately connected.

Clinically and anatomically we note a close and intimate relation existing between the genitals and the nasal mucosa, the larynx (voice) and the sebaceous glands of the face. The whole manifestation is due to reflex action carried on through the sympathetic nerves. The frequent hemorrhage of the nose during and subsequently to puberty in both sexes demonstrates the intimate relation of the nasal mucosa to the generative organs. The vicarious hemorrhage assumed by the nasal mucosa shows the close relation of genitals and nasal mucosa. Again, why is it that so many women we note with chronic uterine disease have rhinitis in different forms? A typical example came to my office a few days ago. She was 24 years old and single. At 20 she began to be irregular in menstrual function, and to have menorrhagia. Digital examination revealed a quite large, hypertrophic, metritic uterus, fixed by old adhesions, with distinct retro-flexion. She said she bled frequently at the nose. The tissue covering the turbinated bones was thickened, inflamed and congested. The chronic rhinitis and metritis coexisted. Many diseased generative organs co-exist with diseased nasal mucosa.

